# Factors Associated With COVID-19 Vaccination Among Individuals With Vaccine Hesitancy in French-Speaking Belgium

**DOI:** 10.1001/jamanetworkopen.2022.34433

**Published:** 2022-09-16

**Authors:** Gloria Yawavi Gbenonsi, Aline Labat, Amandine Oleffe, Boris Jidovsteff, Olivier Servais, Nicolas Vermeulen, Elisabeth Paul

**Affiliations:** 1 School of Public Health, Université libre de Bruxelles, Brussels, Belgium; 2 Research Unit for a Life-Course Perspective on Health and Education, Université de Liège, Liège, Belgium; 3 Prospective Anthropology Lab, Université catholique de Louvain (UCLouvain), Ottignies-Louvain-la-Neuve, Belgium; 4 Psychological Sciences Research Institute, UCLouvain, Brussels, Belgium; 5 Fund for Scientific Research, UCLouvain, Brussels, Belgium

## Abstract

This cross-sectional study examines why people in French-speaking regions of Belgium who initially resisted getting the COVID-19 vaccine eventually chose to get vaccinated.

## Introduction

Despite unprecedented communication campaigns, many governments faced some degree of COVID-19 vaccine hesitancy.^1,2^ In Belgium, French-speaking regions (Brussels and Wallonia) are of particular interest, as their vaccination rates are lower than that in Flanders. In October 2021, the government enacted a Covid Safe Ticket (CST) pass, which conditioned access to several public and private facilities on proof of complete vaccination, recovery, or a negative polymerase chain reaction test. COVID-19 passes have been implemented to fight the epidemic and were moderately effective in incentivizing vaccination, notably in France.^3^ In this study, we examine the reasons people who were initially resistant to the vaccine ended up getting vaccinated.

## Methods

This cross-sectional study used a mixed-methods design to analyze data from a voluntary online survey (December 2021) and followed the STROBE guideline. Residents of French-speaking Belgium involved in this research reported being fully vaccinated against COVID-19 while mistrusting vaccines. We analyzed the reasons reported for getting vaccinated and sociodemographic variables, using a 2-tailed χ^2^ test of independence and a Bonferroni correction, with *P* < .003 considered statistically significant. Participants’ responses to an open-ended question about their thoughts on vaccination were subject to in-vivo coding and thematic qualitative analysis. The self-reported questionnaire followed all ethical recommendations ensuring participants’ protection (eMethods in the Supplement).

## Results

Overall, 918 of 3171 fully vaccinated participants (29%) reported a low confidence level with COVID-19 vaccines. Sociodemographic characteristics of the sample appear in Table 1. Table 2 presents, in decreasing order, the most common reasons given for vaccination despite low confidence. Respondents got vaccinated to facilitate travel (444 [48%]) and recover freedom in day-to-day life (399 [44%]); 387 (42%) reported social pressure. Only 88 (10%) got vaccinated out of personal protection against COVID-19.

**Table 1.** Sociodemographic Characteristics of the Study Population

Characteristic	Respondents, No. (%) (N = 918)
Gender	
Women	582 (63)
Men	334 (36)
Others	2 (1)
Age, y	
18-29	75 (8)
30-44	360 (39)
45-64	404 (44)
>65	78 (9)
Education attainment	
High	698 (76)
Low	160 (17)
Do not wish to respond	60 (7)

**Table 2.** Analysis of the Reasons Reported for Getting Vaccinated by Respondent Gender, Age, and Education Attainment

Reasons evoked	Respondents, No. (%) (N = 918)	Gender^a^			Age, y					Education attainment			
		Women (n = 582)	Men (n = 334)	*P* value	18-29 (n = 75)	30-44 (n = 360)	45-64 (n = 404)	>65 (n = 78)	*P* value		High (n = 698)^b^	Low (n = 160)^c^	*P* value
Facilitate travel or holiday	444 (48.4)	276 (47.4)	168 (50.3)	.40	51 (68.0)	177 (49.2)	184 (45.5)	31 (39.7)	.002		338 (48.4)	80 (50.0)	.71
Recover freedom	399 (43.5)	238 (40.9)	161 (48.2)	.03	46 (61.3)	167 (46.4)	157 (38.9)	29 (37.2)	.001		289 (41.4)	86 (53.8)	.005
Social pressure	387 (42.2)	260 (44.7)	127 (38.0)	.05	41 (54.7)	164 (45.6)	162 (40.1)	20 (25.6)	.001		297 (42.6)	64 (40.0)	.55
Avoid multiple tests when Covid Safe Ticket applies	302 (32.9)	197 (33.8)	105 (31.4)	.45	43 (57.3)	118 (32.8)	125 (30.9)	17 (21.8)	<.001		241 (34.5)	47 (29.4)	.21
End of the crisis or back to normal life	216 (23.5)	149 (25.6)	67 (20.1)	.05	25 (33.3)	85 (23.6)	93 (23.0)	14 (17.9)	.14		157 (22.5)	44 (27.5)	.17
To avoid children’s vaccination	217 (23.6)	161 (27.7)	56 (16.8)	<.001	6 (8.0)	116 (32.2)	80 (19.8)	15 (19.2)	<.001		173 (24.8)	22 (13.8)	.003
To resume my work or studies	178 (19.4)	112 (19.2)	66 (19.8)	.84	34 (45.3)	66 (18.3)	72 (17.8)	6 (7.7)	<.001		146 (20.9)	19 (11.9)	.009
To protect my beloved (at-risk people)	155 (16.9)	109 (18.7)	46 (13.8)	.05	19 (25.3)	63 (17.5)	63 (15.6)	11 (14.1)	.18		111 (15.9)	33 (20.6)	.14
Limit contaminations	138 (15.0)	89 (15.3)	49 (14.7)	.80	7 (9.3)	53 (14.7)	62 (15.3)	17 (21.8)	.19		103 (14.8)	26 (16.3)	.63
Out of solidarity	110 (12.0)	82 (14.1)	28 (8.4)	.01	8 (10.7)	42 (11.7)	48 (11.9)	12 (15.4)	.79		88 (12.6)	19 (11.9)	.80
Own protection against COVID-19	88 (9.6)	55 (9.5)	33 (10.0)	.83	6 (8.0)	30 (8.3)	39 (9.7)	13 (16.7)	.14		66 (9.5)	17 (10.6)	.65
Avoid hospital overcrowding	78 (8.5)	52 (8.9)	26 (7.8)	.54	3 (4.0)	33 (9.2)	35 (8.7)	8 (10.3)	.48		66 (9.5)	9 (5.6)	.12
Professional consciousness	69 (7.5)	53 (9.1)	16 (4.8)	.01	3 (4.0)	28 (7.8)	33 (8.2)	5 (6.4)	.62		62 (8.9)	6 (3.8)	.03
Influence of health professional	36 (3.9)	23 (4.0)	13 (3.9)	.96	0	15 (4.2)	17 (4.2)	4 (5.1)	.32		28 (4.0)	7 (4.4)	.83
Be a person at risk	31 (3.4)	19 (3.3)	12 (3.6)	.79	2 (2.7)	8 (2.2)	15 (3.7)	6 (7.7)	.10		23 (3.3)	7 (4.4)	.50
Know people who had severe COVID-19	11 (1.2)	8 (1.4)	3 (0.9)	.52	1 (1.3)	5 (1.4)	2 (0.5)	3 (3.8)	.09		8 (1.1)	1 (0.6)	.55

^a^ The 2 respondents declared as other gender were considered as missing value.

^b^ High education attainment includes superior (short/long) and third cycle.

^c^ Low education attainment includes secondary, primary, and none.

Sociodemographic variables were associated with some of the reported reasons (Table 2). Women and highly educated people were more likely to report the desire to avoid children’s vaccination. Youths (aged 18-29 years) were more likely to be vaccinated for reasons related to individual liberty (facilitate travel/holiday; recover freedom) and social or professional pressure (Table 2).

The qualitative analysis shows that “recovering freedom” and “escaping government constraints” (travel restrictions and CST) were evoked by most respondents (55 of 86). Moral pressure from close contacts (ie, family, coworkers), the media, and government were mentioned by half of respondents (43). They reported feeling discriminated against and guilty, notably due to continuous recall of the government’s announced vaccination target of 70% supposed to end the pandemic and the suspicion of being an “anti-vaxxer” or “conspiracist” if unvaccinated, while moral pressure was particularly reported in the medical and commercial professions. Some respondents evoked a “social divide” exacerbated by the media and policy makers.

## Discussion

A significant portion of individuals with vaccine hesitancy got vaccinated for various reasons, mostly related to escaping governmental constraints, to moral pressure, and to a collective effort to end the pandemic. Importantly, individual protection against COVID-19 was not the main reported reason, as it was the case in the population of fully vaccinated people who were confident in the vaccine. Age, gender, and education were associated with the reported reasons, suggesting the need to better tailor pandemic response strategies.

Many respondents referred to some form of disguised vaccine obligation. Collectively, negative emotions (guilt) and moral pressure from society appeared to be associated with vaccination willingness, as proposed by others.^2,4^ This suggests that government restrictions were somewhat effective in increasing vaccination willingness.^3,5^ Nevertheless, if the government strategy affected the motivation to vaccinate, it also generated socioemotional and ethical costs mainly related to a feeling of polarization of society among many of our respondents. Given that the sample analyzed was not representative of the population, the results must be interpreted with caution, which is an important limitation.

## Notes

Supplement.
eMethods.
