# Error in Author Affiliation

**DOI:** 10.1001/jamanetworkopen.2022.41036

**Published:** 2022-10-18

In the Research Letter titled “Factors Associated With COVID-19 Vaccination Among Individuals With Vaccine Hesitancy in French-Speaking Belgium,”^1^ published September 16, 2022, there was an error in the author affiliations. The affiliations for Nicolas Vermeulen should have read Psychological Sciences Research Institute, UCLouvain, Louvain-la-Neuve, Belgium, and Fund for Scientific Research, Brussels, Belgium. This article has been corrected.^1^
