# Error in Author Name

**DOI:** 10.1001/jamanetworkopen.2023.4289

**Published:** 2023-03-01

In the Research Letter titled “Factors Associated With COVID-19 Vaccination Among Individuals With Vaccine Hesitancy in French-Speaking Belgium,”^1^ published September 16, 2022, Boris Jidovtseff’s name was misspelled in the byline and Article Information section. This article has been corrected.^1^
