# Supplementary material for: Factors Associated With COVID-19 Vaccination Among Individuals With Vaccine Hesitancy in French-Speaking Belgium
Source: JAMA Netw Open. 2022 Sep 16;5(9):e2234433. doi: 10.1001/jamanetworkopen.2022.34433 (PMC9482060; doi:10.1001/jamanetworkopen.2022.34433)
Supplement: Supplement. — eMethods. [file jamanetwopen-e2234433-s001.pdf]

Supplemental Online Content

Gbenonsi GY, Labat A, Oleffe A, et al. Factors associated with COVID-19 vaccination among individuals with vaccine hesistancy in French-speaking Belgium. *JAMA Netw Open.* 2022;5(9):e2234433. doi:10.1001/jamanetworkopen.2022.34433

eMethods.

This supplemental material has been provided by the authors to give readers additional information about their work.

© 2022 Gbenonsi GY et al. *JAMA Network Open.*

eMethods.

Details of the online survey and the representativeness of French-speaking Belgians

The research is based on a voluntary, self-administered online questionnaire. The questionnaire was developed on the Lime survey platform and was actively disseminated on social networks (mainly Facebook and Twitter) between 4 and 18/12/2021. Several follow-ups were carried out in order to reach the widest possible population. A total of 9830 people took part in the survey. After cleaning out outliers and insufficiently complete data, we retained a database of 9444 respondents on which analyses were performed. The sample surveyed was not representative of the French-speaking Belgian population. 3,171 participants declared to be fully vaccinated against COVID-19 (807 with a booster dose). Among them 918 (29%) reported to mistrust the COVID-19 vaccines. This specific subgroup constitutes the sample of our study population.

Measures used for the study

Reasons for vaccination were proposed to participants through the study's closed question: "Which of the following really prompted you to get vaccinated?

The question was multiple choice and 16 non-exclusive answers were suggested:

1) To protect myself against COVID-19
2) To limit contaminations and the epidemic
3) To travel, go on holiday
4) Out of social pressure (work, school, family...)
5) To resume my work/studies
6) To get out of the crisis and be back to normal
7) To protect my beloved (at-risk people)
8) Because a health professional convinced me to do so
9) Because I am at risk
10) To be free and avoid constraint (leisure, sport, avoid tests, lift masks...)
11) Out of professional consciousness
12) Because I know people deceased from COVID-19 and I am scared
13) To avoid children vaccination
14) Not to have to make a test where CST is imposed
15) To reduce hospital pressure
16) Out of solidarity

A two-tailed chi-square test of independence was carried out to analyze the association between the different reasons identified and the profile of the respondents. As 16 Chi-square tests were performed

© 2022 Gbenonsi GY et al. *JAMA Network Open.*

for each explicative variable (gender, age and education attainment), a Bonferroni correction was applied, leading to a level of significance of 0,003 for all the p-values presented (0,05/16=0,003). A two-tailed test offers the possibility of concluding whether or not there is an association between the variables studied without, however, determining the direction of this association, especially when the categories compared are greater than two.

Categorization of participants according to their level of confidence

To categorize respondents according to their level of confidence, we used the question "How confident are you in covid-19 vaccines?"

The response modalities ranged from 'very low- 1' to 'very high-9'. Thus, individuals were classified as having a low level of confidence when they chose answers 1, 2 or 3.

Method of the qualitative component

The qualitative analysis was based on the answers given to one of the four open questions at the end of the questionnaire: "What do you think about vaccination?". The answers were coded using the "in-vivo" method that uses the respondents' own vocabulary and we then carried out an analysis using the phenomenological approach. In total, 3 persons (YGG, EP and Morgane Guyomarch) were involved in the coding and qualitative analysis. We found a consensus when the codes were different.

Ethical precautions

Regarding the ethical rules, we pay particular attention to respect the Helsinki agreements, comprising: informed consent, voluntary participation to the study, freedom to quit the questionnaire at any time without constraint and without justification, ability to erase all their data, confidentiality, completely anonymous (The data file does not contain any names or email addresses that could be used to identify the participants, the IP address was requested to avoid duplicates and was erased from the shared analysis file, making the data anonymous), and the presence at the end of the questionnaire of a specific email address in case of questions (qcovid19@uliege.be).

© 2022 Gbenonsi GY et al. *JAMA Network Open.*
